# What is new in hemodynamic monitoring and management?

**DOI:** 10.1007/s10877-022-00848-8

**Published:** 2022-04-08

**Authors:** Moritz Flick, Alina Bergholz, Pawel Sierzputowski, Simon T. Vistisen, Bernd Saugel

**Affiliations:** 1 Department of Anesthesiology, Center of Anesthesiology and Intensive Care Medicine, University Medical Center Hamburg-Eppendorf, Hamburg, Germany; 2 Department of Anaesthesiology and Intensive Care, Aarhus University Hospital, Aarhus, Denmark; 3 Outcomes Research Consortium, Cleveland, Ohio USA

**Keywords:** Blood pressure, Cardiac output, Hemodynamic monitoring, Hemodynamic management, Cardiovascular dynamics, Artificial intelligence

## Introduction

Hemodynamic monitoring and management are essential pillars of perioperative care. Innovative continuous and non-invasive monitoring solutions require profound validation before they may improve perioperative care. New monitoring tools are changing how we monitor and manage hemodynamics in surgical and critically ill patients and many of these are published in the Journal of Clinical Monitoring and Computing (JCMC). In this What-is-new review, we will highlight some of the papers focusing on what is new in the area of hemodynamic monitoring and management based on papers published in the JCMC in 2020 and 2021.

## Blood pressure monitoring

Meidert et al. [1] conducted a prospective observational study comparing intermittent oscillometric blood pressure measurements with continuous invasive radial artery blood pressure measurements in 30 hypotensive patients admitted to the resuscitation area of an emergency department. The authors defined hypotension as an invasive radial arterial mean arterial pressure <60 mmHg. Seventy-five oscillometric and invasive blood pressure measurement pairs were analyzed using Bland-Altman analysis. The mean of the differences between oscillometric and invasive radial arterial mean arterial pressure measurements was 13 mmHg (95%-limits of agreement -16 to 41 mmHg). Interestingly, 48 of 75 (64%) oscillometric measurements showed a mean arterial pressure ≥60 mmHg and, thus, did not detect hypotension. The authors conclude that oscillometric blood pressure monitoring is not able to accurately detect hypotension in patients treated in the emergency department. Consequently, arterial catheters for invasive continuous blood pressure monitoring should be inserted as soon as possible. These findings are in line with other studies investigating the accuracy of oscillometric blood pressure measurements compared to invasive reference measurements [2, 3] and emphasize the importance of knowing typical pitfalls and limitations of oscillometric blood pressure monitoring.

In a retrospective cohort study, Yoon et al. [4] investigated the relationship between intraoperative blood pressure stability and postoperative mortality in 1203 patients having cardiac surgery with cardiopulmonary bypass. The authors hypothesized that intraoperative blood pressure stability based on individual preoperative blood pressure measurements could predict postoperative mortality. Intraoperative blood pressure stability was determined by calculating performance measurement variables, i.e. median performance error, median absolute performance error, and wobble using preoperative baseline blood pressure measurements as reference value [5]. There was no difference in baseline blood pressure measurements between 30-day survivors (n = 1175) and non-survivors (n = 28). A total of 108,698 intraoperative mean arterial pressure measurements were included in the analysis. Mean arterial pressure was lower in non-survivors. For analysis of blood pressure stability, a higher median absolute performance error and lower median performance error were observed in non-survivors compared with survivors. Non-survivors showed significant time-dependent variance in mean arterial pressure, which was accompanied by a higher wobble, compared with survivors during cardiopulmonary bypass. After adjusting for confounding variables, median performance error and median absolute performance error were independent predictors of short and long-term postoperative mortality. The authors suggest that calculation of performance measurement variables is a valid method to quantify blood pressure stability during surgery and to predict postoperative mortality. Analyzing blood pressure stability is difficult and not standardized, but may play an important role in the investigation of hemodynamic treatment and protocol adherence in goal-directed therapy trials.

In clinical routine, blood pressure is routinely measured non-invasively using upper-arm cuff oscillometry. However, it remains unknown whether brachial blood pressure adequately reflects aortic blood pressure – or if there are substantial differences. Chemla et al. [6] performed a systematic review of studies reporting both, blood pressure values from the aorta and brachial artery. The authors aimed to investigate differences in all three blood pressure components, systolic arterial pressure, diastolic arterial pressure, and mean arterial pressure, between the peripheral and the aortic blood pressure. The authors identified six studies with a total of 294 adult awake patients, mostly during heart catheterization for suspicion of coronary artery disease. Heterogeneity in recorded blood pressure components did not allow reporting pooled results for all studies. Two studies reported all blood pressure components (n = 64 patients). The pooled absolute (relative) blood pressure difference for systolic arterial pressure was 4.2 mmHg (3.1%), for mean arterial pressure 0.1 mmHg (0.1%), and for diastolic arterial pressure -1.3 mmHg (-1.8%) in these two studies. Four studies only reported systolic and diastolic arterial pressure (n = 230 patients). The pooled absolute (relative) blood pressure difference for systolic arterial pressure was 6.6 mmHg (4.9%) and for diastolic arterial pressure 0.2 mmHg (0.3%) in these four studies. Overall, these results indicate minor differences between brachial and aortic blood pressure. Clinicians can thus consider brachial blood pressure measurements as a reliable approximation of aortic blood pressure – but should consider that brachial systolic pressure may be higher, e.g. due to reflection phenomena.

Joachim and colleagues [7] investigated whether beat-to-beat mean arterial pressure values can be derived from photoplethysmographic finger measurements. The authors used different algorithms considering photoplethysmography-derived variables (notch relative amplitude, notch absolute amplitude, perfusion index) to derive beat-to-beat mean arterial pressure values in a retrospective analysis of 46 surgical patients. The calibration of photoplethysmography-derived mean arterial pressure values was intermittently performed using oscillometric mean arterial pressure measurements. Estimated mean arterial pressure values were then compared to oscillometric mean arterial pressure measurements using Bland-Altman analysis and the mean of the differences with corresponding 95%-limits of agreement was calculated. Estimated mean arterial pressure values were also compared to invasive mean arterial pressure measurements calculating the mean absolute error with interquartile ranges. Mean arterial pressure values of the most accurate prediction algorithm – that uses the absolute notch amplitude of the photoplethysmography curve – showed a mean of the differences (95%-limits of agreement) of -1 (-10 to 8) mmHg compared to oscillometric mean arterial pressure measurements and a mean absolute error (interquartile range) of 11 (5 to 18) mmHg compared to invasive mean arterial pressure measurements. The authors conclude that continuous estimation of photoplethysmography-derived beat-to-beat mean arterial pressure values during general anesthesia appears possible with an acceptable average error. It will be interesting to see if photoplethysmography will allow reliable non-invasive continuous blood pressure monitoring. If possible, it may become a valuable tool for postoperative monitoring on normal wards, where other methods are usually not available.

## Cardiac output monitoring

Pulse wave analysis is a well-established method for continuous cardiac output monitoring based on an invasive or non-invasive blood pressure waveform [8]. Nonetheless, there are substantial differences between different pulse wave analysis algorithms and their underlying principles [9].

In two recent method comparison studies, the measurement performance of a pulse wave analysis monitor using the multi-beat analysis approach (Argos cardiac output monitor, Retia Medical; Valhalla, NY, USA) to estimate cardiac output was investigated [3, 4]. First, a study in 58 patients having off-pump coronary artery bypass surgery compared multi-beat analysis cardiac output measurements to pulmonary artery thermodilution reference cardiac output measurements [10]. A total of 572 paired cardiac output measurements were analyzed using Bland-Altman analysis, percentage error, and four-quadrant plots. Radial arterial blood pressure waveforms were retrospectively fed into the device for offline cardiac output estimation using multi-beat analysis. The mean of the differences (95%-limits of agreement) between multi-beat analysis cardiac output and pulmonary artery thermodilution cardiac output was -0.2 (-2.5 to 2.1) L/min with a percentage error of 51%. The concordance rate to track ≥15% cardiac output changes was 89% between multi-beat analysis and pulmonary artery thermodilution. The second study also compared multi-beat analysis cardiac output measurements from the Argos cardiac output monitor with pulmonary artery thermodilution cardiac output measurements in 31 patients treated in the intensive care unit after off-pump coronary artery bypass surgery [11]. A total of 167 cardiac output measurement pairs were compared using Bland-Altman analysis, percentage error, and four-quadrant plots. The mean of the differences (95%-limits of agreement) between multi-beat analysis cardiac output and pulmonary artery thermodilution cardiac output was 0.1 (-2.1 to 2.3) L/min with a percentage error of 41%, The concordance rate in the four-quadrant plot was 88%. Just as in the first study – from the same research group – the radial arterial blood pressure waveforms were retrospectively transferred into the device for offline cardiac output estimation using multi-beat analysis. Both studies show that the agreement between multi-beat analysis cardiac output and pulmonary artery thermodilution cardiac output is not clinically acceptable (when defining clinically acceptable agreement as a percentage error of less than 30%).

Transthoracic echocardiography is often used for bedside hemodynamic assessment. Transthoracic echocardiography also allows measuring cardiac output. In a prospective method comparison study in 50 patients one day after cardiac surgery, Juhl-Ohlsen et al. [12] compared both automated echocardiography cardiac output (Auto VTI Tool® - Venue R1 ultrasound system; GE Healthcare, Horten, Norway) and manual echocardiography cardiac output with continuous pulmonary artery thermodilution cardiac output (reference method). Automated and manual echocardiography cardiac output measurements were performed in a randomized order to prevent bias. For comparison, five averaged pulmonary artery thermodilution cardiac output readings were averaged. The mean of the differences (95%-limits of agreement) between automated echocardiographic and pulmonary artery thermodilution cardiac output was 0.7 (-1.9 to 3.3) L/min with a percentage error of 46%. The concordance rate to track ≥10% cardiac output changes was 47%. The mean of the differences (95%-limits of agreement) between manual echocardiographic and pulmonary artery thermodilution cardiac output was 0.7 (-1.9 to 3.2) L/min with a percentage error of 44% and a concordance rate of 44%. Neither automated nor manual echocardiography thus allowed clinically acceptable cardiac output measurements – which is somewhat disappointing. Further, the trending ability – the ability to follow cardiac output changes – was poor. This study underlines that cardiac output measurements with echocardiography need to be interpreted with caution, but echocardiography is nonetheless an important method for hemodynamic assessment – especially in patients with circulatory shock.

Non-invasive cardiac output monitoring remains challenging. The cardiac output measurement performance of electrical cardiometry (which was developed from bioimpedance) had not been systematically evaluated in a review and meta-analysis. Sanders et al. [13] thus performed a systematic review and meta-analysis of 13 studies with 620 adult patients and 11 studies with 603 pediatric patients. The inter-study heterogeneity was high – including different reference cardiac output methods. In adults, the pooled mean of the differences between electrical cardiometry and reference cardiac output was 0.03 L/min with pooled 95%-limits of agreement of -2.78 to 2.84 L/min and a pooled percentage error of 48%. In pediatric patients, the pooled mean of the differences was -0.02 L/min with pooled 95%-limits of agreement of -1.22 to 1.18 L/min and a percentage error of 42%. The pooled percentage errors indicate that the agreement between electrical cardiometry and reference cardiac output is not clinically acceptable in both adults and children. Pooled percentage errors are similar to those reported for other non-invasive cardiac output monitoring methods in previous meta-analyses, e.g. 43% for finger cuff methods [14], 45% for partial carbon dioxide rebreathing [15], and 43% for transthoracic electrical bioimpedance [15].

In an experimental study with 8 pigs having ventilator-induced lung injury, Sigmundsson et al. [16] compared cardiac output measurements using the capnodynamic method with cardiac output measurements using an ultrasonic flow probe around the pulmonary trunk. The measurement principle of the capnodynamic method has been previously described in detail [17]. Lung injury was induced with repeated lung lavages and harmful mechanical ventilation. Measurements were performed at baseline without lung injury and after induction of lung injury, at a positive end-expiratory pressure of 5 cmH_2_O and at an adjusted positive end-expiratory pressure of 11–17 cmH_2_O. At baseline, the mean of the differences (95%-limits of agreement) between capnodynamic cardiac output and flow probe cardiac output was 0.5 (-0.5 to 1.5) L/min with a percentage error of 30%. After induction of lung injury with a positive end-expiratory pressure of 5 cmH_2_O, the mean of the differences was -0.6 (-2.3 to 1.1) L/min with a percentage error of 39%. With an adjusted positive end-expiratory pressure, the mean of the differences was 1.1 (-0.3 to 2.5) L/min with a percentage error of 38%. The concordance rate to track changes in cardiac output ≥10% caused by temporary balloon inflation in the vena cava to reduce preload and dobutamine infusion was 87% at a positive end-expiratory pressure of 5 cmH_2_O and 100% with adjusted positive end-expiratory pressure levels. Even though this was a small experimental study, it shows that estimating cardiac output using the capnodynamic method – even during respiratory failure – is possible, but did not show clinically acceptable agreement compared to flow probe cardiac output. Flow probe cardiac output is considered the gold standard for cardiac output measurements, but rarely feasible outside of experimental studies. It will be interesting to see how the capnodynamic method performs in clinical studies.

Most studies on hemodynamic monitoring focus on monitoring in operating rooms and intensive care units. Monitoring vital signs, especially blood pressure, non-invasively and continuously on normal wards remains challenging but may be an important step to reduce postoperative mortality [18]. In a prospective observational study, King et al. [19] investigated the feasibility of continuous non-invasive blood pressure and advanced hemodynamic monitoring using the finger sensor technology LiDCO CNAP (LiDCO; London, United Kingdom) during the first 12 postoperative hours in 104 patients treated in the post-anesthesia care unit and on the normal ward after non-cardiac surgery. The primary aim was to investigate whether postoperative finger sensor monitoring is feasible. In only 41 patients (39%), continuous monitoring was performed throughout the 12-hours period. Most common reasons for discontinuation of monitoring were patient discomfort in the fingers, in the upper arm (from oscillometric recalibration), and technical problems. Postoperative hypotension, defined as a systolic arterial pressure <90 mmHg, occurred in 27 patients (26%) in the post-anesthesia care unit and in 46 patients (48%) on the normal ward. The mean (± standard deviation) total duration of hypotension – in patients with at least one hypotensive episode – was 27.4 (± 24.7) min in the post-anesthesia care unit and 82.7 (± 97.2) min on the normal ward. Not surprisingly, hypotension was rarely detected by intermittent routine care monitoring. Interestingly, low systemic vascular resistance was present in 44% of hypotensive episodes suggesting that vasodilation is a common cause of postoperative hypotension. Treating postoperative hypotension pragmatically with fluids – as vasopressors are rarely available on normal wards – may thus not always be the optimal therapy. This study provides important information on the incidence and underlying causes of postoperative hypotension and underlines some of the challenges that need to be overcome to establish continuous ward monitoring in routine care [20, 21].

In a retrospective analysis of a prospectively obtained database, Herner et al. [22] investigated effects of veno-venous extracorporeal membrane oxygenation (ECMO) on transpulmonary thermodilution-derived variables. Specifically, the aim was to determine if potential loss of indicator fluid – caused by ECMO – leads to falsely high volumetric variables. Transpulmonary thermodilution data of 14 patients with severe acute respiratory distress syndrome treated with veno-venous-ECMO were available for analysis. Mean cardiac index (± standard deviation) was 4.5 ± 1.7 L/min/m^2^ before and 4.4 ± 2.1 L/min/m^2^ (p = 0.43) after initiation of ECMO therapy. After initiation of ECMO therapy, extravascular lung water index increased from 21 ± 9 mL/kg to 28 ± 11 mL/kg (p < 0.01), and global end-diastolic volume index increased from 791 ± 179 mL/m^2^ to 974 ± 384 mL/m^2^ (p = 0.04) compared to before initiation of ECMO therapy. The authors provide a list of ten recommendations for hemodynamic monitoring before and during ECMO therapy.

## Peripheral perfusion index monitoring

It is assumed that systemic hemodynamics – reflected by cardiac output or blood pressure – are coupled with microcirculatory tissue perfusion. The peripheral perfusion index is a photoplethysmography-derived microcirculatory tissue perfusion variable that has been associated with impaired outcome in surgical patients [23, 24].

In a prospective observational study, Højlund and colleagues [25] investigated the ability of the peripheral perfusion index to detect changes in stroke volume, cardiac output, and mean arterial pressure in 20 abdominal surgery patients during the post-induction period. The peripheral perfusion index was measured continuously and non-invasively using photoplethysmography. The authors compared changes in the peripheral perfusion index with changes in stroke volume, cardiac output, and mean arterial pressure while tilting the patient table with different maneuvers (head-up tilt, head-down tilt, and head-up tilt with simultaneous phenylephrine administration). Spearman’s rank correlation (95% confidence interval) was used to evaluate the relationship between relative changes in peripheral perfusion index and relative changes in stroke volume, cardiac output, and mean arterial pressure. The results showed a strong correlation between changes in peripheral perfusion index and changes in stroke volume r = 0.9 (95%-confidence interval 0.7-1.0), in cardiac output r = 0.9 (0.5-1.0), and in mean arterial pressure r = 0.9 (0.7-1.0) (p < 0.001 each). The authors conclude that changes in the peripheral perfusion index thus well reflect changes in hemodynamic variables during preload-changing maneuvers in patients under general anesthesia.

De Courson et al. investigated whether changes in perfusion index reflect changes in stroke volume during lung recruitment maneuvers in 47 patients having neurosurgery [26]. Peripheral perfusion index was monitored continuously using a standard pulse oximeter. Additionally, stroke volume index was monitored using pulse wave analysis of the radial arterial catheter-derived blood pressure waveform. Lung recruitment maneuver was performed by applying 30 cmH_2_O for 30 s. If stroke volume index decreased by >30% during the lung recruitment maneuver patients were considered fluid responsive and a 250 mL fluid challenge was performed over 10 min [27]. Twenty-four out of 47 patients were considered fluid responsive with a decrease in mean ± standard deviation stroke volume index from 39 ± 7 mL/m^2^ to 24 ± 4 mL/m^2^ with a simultaneous decrease in perfusion index from 5.9 ± 3.1% to 3.8 ± 2.4%. In fluid unresponsive patients, the change in mean stroke volume index was 39 ± 11 mL/m^2^ to 33 ± 9 mL/m^2^ with a simultaneous decrease in perfusion index from 7.6 ± 4.4% to 6.2 ± 3.5%. Pearson’s correlation coefficient between changes in stroke volume index and perfusion index was r^2^ = 0.34. In the 24 patients considered fluid responsive, fluid boluses increased mean stroke volume index by 16% and perfusion index by 17% - with a weak correlation (r^2^ = 0.19). The authors conclude that monitoring the perfusion index – especially during lung recruitment maneuvers – is a simple and non-invasive method to detect fluid responsiveness. It may therefore provide important information when stroke volume monitoring is not indicated – or as an additional tool.

These studies show the vast potential of measuring the peripheral perfusion index. A recent retrospective study further showed a strong correlation between impaired intraoperative peripheral perfusion index and postoperative outcomes [23]. With growing evidence showing the importance of the peripheral perfusion index, it will be interesting to see how photoplethysmography and the peripheral perfusion index can be integrated into hemodynamic monitoring and management.

## Artificial intelligence

An interesting study on artifact detection in vital signs time series was conducted by Pasma et al. [28]. In the study, two trained research assistants observed in a live setting the arterial blood pressure waveform for at least one hour in 88 surgical procedures and annotated artifacts. In addition, artifacts during the same procedures were retrospectively annotated by a third person based on recorded data, i.e. without the full clinical context. Furthermore, three machine learning algorithms made use of the same standard monitoring time-series data (mean, diastolic and systolic pressures, and heart rate) and a number of features derived thereof. The main findings were (1) that retrospective artifact annotations only had a 32% sensitivity in identifying the annotations made in the live setting, and (2) that machine learning algorithms did not provide better performance. The authors concluded that the overall performance remained moderate. It is not new that advanced algorithms have trouble in annotating artifacts, but the most important finding of the study is probably that “it is not only important to describe who annotated data, but also when and how data points were marked as artifacts, in order to make research reproducible” [28]. Automated or manual artifact rejection in the retrospective analysis of large datasets may be prone to a similar, unsolvable artifact issue, because information about the clinical context is not recorded in most datasets available for retrospective analysis.

A study published in 2020 by Keim-Malpass et al. [29] investigated alert thresholds for clinical deterioration. These authors correctly recognize in their introduction that there is a huge gap between the development of a predictive algorithm based on a retrospective dataset and its successful adoption into bedside clinical care. Their study was nonetheless a retrospective analysis of patient data obtained from a cardiac acute care ward. The authors implemented a case-control design with propensity score matching and they made use of 8111 adults’ admission data, with an approximate split of 50%/50% between the training and test data. The authors reported that so-called *risk spikes* could predict 7.3% of intensive care unit transfers with a positive predictive value of 7.4%, which may be a fair but not an outstanding result. Risk spikes were introduced as they may serve as a correction for a patient’s own baseline physiology, which scores such as the National Early Warning Score (NEWS) do not. Unfortunately, the paper did not report and discuss detailed results of a reference model/predictor, such as the NEWS score’s performance. The authors made a propensity score matching which may be better than a more random selection of non-events. Still, a subsequent clinical implementation of the risk spike algorithm is likely to perform dramatically different from the results obtained with a case-control design, because the case-control design applied to time-series is inherently contributing a temporal bias in the analysis [30]. Applying such a design is therefore unlikely to bridge the gap between a retrospectively developed algorithm and real-world clinical implementation [31].

In 2021, JCMC also published a highly interesting study investigating closed-loop (CL) resuscitation in an experimental animal (pig) model of hemorrhagic shock [32]. Anesthetized pigs were bled to a mean arterial pressure level between 30 and 35 mmHg and maintained there for 90 min, after which interventions were made. The study presented results of two series of experiments, where the protocol 1 experiment (n = 20) had three post-bleeding treatment arms: A clinician resuscitating manually with fluids, a CL algorithm administering the resuscitation with fluids, and a CL algorithm administering a combination of fluids and norepinephrine. In the protocol 2 experiment (n = 24), only the CL treatment arm was investigated. There was an exclusively-fluid arm and two fluid + norepinephrine arms where high and moderate doses of norepinephrine, respectively, were administered. Also, the second experiment made use of a discontinuous measurement of blood pressure, whereas the first experiment used continuous blood pressure monitoring. The main finding of this study was that the CL treatment arms did not result in statistically significant differences between the groups in terms of reaching and maintaining the treatment target set up for resuscitating the animals. An additional important result was that the CL algorithms worked best when a continuous blood pressure signal was available. Finally, the authors also found that pigs resuscitated with norepinephrine required less fluid and had less hemodilution than pigs resuscitated with fluid alone, which was not surprising. It would be fair to say that the study might not have a very high statistical power to make firm conclusions about the non-difference between the treatment groups, and also that the applied model of hemorrhagic shock is not comparable with a clinical setting in many aspects. It should also be noted that the different treatment arms had very different effects on hemodynamics. Yet, the authors are fully aware of this and should be commended for their nuanced discussion of their results. Despite such limitations, the authors clearly demonstrated the overall proof-of-concept for their CL algorithms.

## Hemodynamic outcome trials

Reducing myocardial injury during acute myocardial infarction remains a major medical challenge. It has been suggested that glucose-insulin-and-potassium infusion may reduce myocardial injury during acute myocardial infarction. The results from studies investigating possible protective effects of glucose-insulin-and-potassium infusion vary substantially [33].

Licker et al. [34] performed a randomized parallel group, superiority trial to assess the effects of pre-treatment with glucose-insulin-and-potassium infusion on left ventricular function in 100 moderate-to-high risk patients undergoing on-pump coronary artery bypass graft with or without aortic valve replacement. Patients were randomized to receive either glucose-insulin-and-potassium solution or normal saline before cardiopulmonary bypass. All caregivers were blinded to group allocation. The primary outcomes were two-dimensional left ventricular ejection fraction (2D-LVEF) and three-dimensional left ventricular ejection fraction (3D-LVEF) and transmitral flow propagation velocity. Measurements were performed at three time points: at the start of surgery before glucose-insulin-and-potassium infusion, 20 min after infusion but before aortic clamping, and at the end of surgery. A total of 224 patients were randomized and 100 were included in the final analysis, 46 receiving saline and 54 glucose-insulin-and-potassium. Most patients were excluded because no bypass grafting was performed. At the start of surgery, patients receiving glucose-insulin-and-potassium had lower mean (± standard deviation) 3D-LVEF compared to patients receiving saline (42 ± 6% vs. 47 ± 7%); 2D-LVEF was similar between groups (41 ± 6% vs. 43 ± 5%). After infusion of glucose-insulin-and-potassium, 3D-LVEF was 44 ± 6% and 2D-LVEF was 41 ± 6% before aortic clamping and did not change until the end of surgery (3D-LVEF: 44 ± 6%; 2D-LVEF 42 ± 6%). In patients receiving saline, 3D-LVEF was 44 ± 7% and 2D-LVEF was 40 ± 6% before aortic clamping and 41 ± 8% (3D-LVEF) and 39 ± 8% (2D-LVEF) at the end of surgery. These changes indicate a minor improvement in left ventricular ejection fraction in patients receiving glucose-insulin-and-potassium, whereas patients receiving saline had a decreased left ventricular ejection fraction at the end of surgery. Further, the authors report that glucose-insulin-and-potassium infusion was associated with a lesser need for cardiovascular drug support, fewer respiratory complications, and shorter intensive care unit length of stay. Further research is necessary to investigate whether the observed differences in left ventricular ejection fraction are clinically important – and whether they translate into improved outcomes.

Perioperative goal-directed therapy, especially cardiac output-guided goal-directed therapy may improve patient outcomes in high-risk non-cardiac surgery [35]. In a before and after trial, Boekel et al. [36] investigated (1) the effect of implementation of a perioperative goal-directed therapy protocol and (2) the compliance to the protocol on postoperative outcomes (defined using the “Expanded Accordion Severity Classification Model” [37]) in 402 patients after high-risk non-cardiac surgery. Data before implementation of the goal-directed therapy protocol (n = 214) were collected retrospectively, whereas data after implementation of the goal-directed therapy protocol (n = 188) were collected prospectively. The implementation of the goal-directed therapy protocol reduced the number of postoperative complications (414 before vs. 282 after implementation of the goal-directed therapy protocol (p = 0.031), especially of severe complications (95 before vs. 47 after implementation of the goal-directed therapy protocol (p = 0.003). Out of 188 available patients treated with the goal-directed therapy protocol, high compliance defined as ≥85% of time within target ranges was achieved in 79 patients (42%). In patients with high protocol compliance, fewer postoperative complications were observed (90 with high vs. 187 with low compliance; p = 0.01). There was no difference in postoperative death between low and high compliance groups. Additionally, the compliance to secondary cardiac index targets was investigated but showed no clinically important differences between high and low compliance. Although a protocolized treatment strategy can improve patient outcomes in general, this study underlines the importance of high protocol compliance. How a high goal-directed therapy protocol compliance can be achieved – especially in high-risk patients in whom several problems may be present simultaneously will need further investigation.

Intraoperative hypotension, although still not clearly defined, is associated with postoperative cardiac and renal morbidity and mortality [38]. Sponholz et al. [39] performed a randomized, open-label trial to investigate the effect of processed electroencephalographic (pEEG; Narcotrend, Hannover, Germany) monitoring-guided anesthesia on norepinephrine requirement in 245 patients having cardiac surgery. Patients were randomized to pEEG-guided anesthesia (Narcotrend Index between 37 and 64) or routine care with blinded pEEG. The primary endpoint was intraoperative norepinephrine requirement. The authors used robust regression including the type of surgery as a covariate. Secondary endpoints were intraoperative fluid balance, time to extubation, postoperative delirium, and intraoperative adverse events. In both groups, a mean arterial pressure between 65 and 85mmHg was targeted. Data of 239 patients were used to analyze the primary endpoint (pEEG-guided: n = 120; routine care: n = 119). Patients in the pEEG-guided group had a robust mean intraoperative norepinephrine requirement of 4.71 µg/kg, which was significantly lower than 6.14 µg/kg in the routine care group. In line with this, vasodilative agents (urapidil and nitroglycerine) were used more frequently in the pEEG-guided group to treat high blood pressure (mean arterial pressure >85mmHg). However, no detailed blood pressure data are presented. In the secondary endpoints, no significant differences in the amount of administered crystalloids and transfused blood products, median time of postoperative ventilation time, and incidence of postoperative delirium were observed between the two groups. In eleven pEEG-guided patients and in two routine care patients, adverse events were reported during anesthesia including unexpected increases of the Narcotrend Index with hypertension, patient movement, coughing, breathing, or perspiration. One patient in the blinded pEEG group experienced awareness. Based on their findings, the authors concluded that individualizing anesthesia depth, e.g. based on pEEG, could be a useful tool to reduce intraoperative hypotension.

The renal resistive index is defined as the difference of the peak systolic and end-diastolic blood velocity divided by the peak systolic velocity measured by Doppler ultrasound in kidney arteries. The renal resistive index is used to evaluate vascular and parenchymal renal abnormalities, but growing evidence indicates that it may also reflect systemic vascular properties [40]. Giustiniano et al. [41] investigated whether renal resistive index, alone or combined with other variables, i.e., complexity and time of the surgical procedure, and postoperative serum lactate clearance, can predict postoperative complications in 183 patients undergoing liver resection in a prospective observational study. Renal resistive index measurements were performed preoperatively and within one hour after emerging from general anesthesia. Postoperative complications were recorded until 7 days after surgery and graded according to the Clavien-Dindo classification. Forty-two out of 183 (22.9%) patients had at least one postoperative complication. Patients with a postoperative complication had a mean (± standard deviation) preoperative renal resistive index 0.66 ± 0.07 compared to 0.61 ± 0.09 observed in patients without postoperative complication. Univariable regression analysis between the renal resistive index and postoperative complications showed an odds ratio of 1.08 (95%-confidence interval 1.02–1.13) for the renal resistive index. As a median value preoperative renal resistive index >0.63 allowed to discriminate a complicated from a non-complicated outcome. Furthermore, multivariable analysis showed that other variables: i.e., high surgical invasiveness, surgery time >360 min, and postoperative serum lactate clearance <-6% were associated with postoperative complications. Considering all the above-mentioned variables, the authors proposed a new scoring system, a complicated outcome prediction score (COPS), ranging from 5 to 16.5 points, which allows stratifying patients into low (COPS 5–10), medium (COPS 10–12), and high (COPS >12) risk groups of postoperative complication. Additionally, a new scoring system was compared with Surgical Apgar Score and Physiological and Operative Severity Score for the enUmeration of Mortality and Morbidity (POSSUM), but rather scant information was provided, that compared to POSSUM, COPS showed no clinically important difference in the area under the receiver operating characteristics curve. Although the presented results are interesting they warrant further investigation, especially to address the question of why a normal preoperative renal resistive index may be associated with postoperative complications.

## Conclusions

This review summarizes new scientific findings in the area of hemodynamic monitoring and management published in the last two years in the JCMC. The JCMC is glad to provide a platform for new research and evidence in this field. More trials investigating how these new methods and technologies for hemodynamic monitoring can improve outcome of surgical and critically ill patients are needed.
